# Glycaemic control in type 1 diabetes mellitus among children and adolescents in a resource limited setting in Dar es Salaam - Tanzania

**DOI:** 10.1186/s12902-016-0113-y

**Published:** 2016-05-31

**Authors:** Mariam Noorani, Kaushik Ramaiya, Karim Manji

**Affiliations:** Aga Khan Hospital, PO Box 2289, Barrack Obama Drive, Dar-es-Salaam, Tanzania; Hindu Mandal Hospital, PO Box 581, Chusi Street, Dar-es-Salaam, Tanzania; Muhimbili University of Health and Allied Sciences, PO Box 65001, UN Road, Dar-es-Salaam, Tanzania

**Keywords:** Type 1 diabetes mellitus, Children, Adolescents, Tanzania, Glycaemic control, Adherence

## Abstract

**Background:**

Type 1 Diabetes Mellitus is a rapidly growing problem in Tanzania. Children and adolescents with type 1 diabetes have previously been found to have poor glycaemic control and high prevalence of complications. Strict glycaemic control reduces the incidence and progression of chronic complications. The aim of this study was to identify the factors associated with glycaemic control among children and adolescents.

**Methods:**

A cross sectional study was done at the diabetes clinic for children and adolescents. Data on socioeconomic, demographic and diabetes specific variables including adherence, diabetes knowledge, caregivers knowledge and their involvement in the care of the child was obtained. Glycaemic control was assessed by measuring glycosylated hemoglobin. (HbA1C). Linear regression analysis was done to determine factors associated with glycaemic control

**Results:**

Seventy-five participants were recruited into the study (51 % males). The mean HbA1c was 11.1 ± 2.1 %. Children aged <10 years were found to have a significantly better glycaemic control (9.8 %) as compared to 10–14 year olds (11.5 %) and >14 year olds (11.4 %) (*P* value = 0.022). Sixty-eight percent of patients had good adherence to insulin while adherence to blood glucose monitoring regimen was 48 % and to diet control was 28 %.

Younger age, having the mother as the primary caregiver, better caregiver knowledge of diabetes, better adherence to blood glucose monitoring regimen and diabetes duration of less than 1 year were associated with better glycaemic control.

In multivariate analysis, age, adherence to blood glucose monitoring regimen and the mother as the primary caregiver were found to independently predict glycaemic control (R^2^ = 0.332, *p* value = 0.00).

**Conclusions:**

Children and adolescents with type 1 diabetes in Dar es Salaam have poor glycaemic control. In order to improve metabolic control, adherence to blood glucose monitoring should be encouraged and caregivers encouraged to participate in care of their children especially the adolescents.

## Background

Type 1 diabetes mellitus (T1DM) is a rapidly growing problem in Tanzania and Africa as a whole. The incidence in some African countries was found to range between 4.4/100,000 in Algeria to 20/100,000 in Morocco. [1] In sub Saharan Africa, few studies have been done but estimates from Sudan showed an increased incidence from 9.5/100,000 in 1991 to 10.3/100,000 in 1995. In Tanzania, the incidence was estimated to be 1.5/100,000 per year in 1991 [2].

In Dar es Salaam, a high prevalence of chronic complications has been described. The prevalence of retinopathy was 22 % and that of micro-albuminuria, which is a marker of nephropathy, was 29 % [3].

As demonstrated in the Diabetes Control and Complication Trial, (DCCT) strict glycaemic control has been shown to reduce the long term complications of type 1 diabetes [4]. Recommendations from the DCCT were to maintain the glycosylated hemoglobin (HbA1c) below 7 %. However, due to the risk of hypoglycemia unawareness in children, the American Diabetes Association (ADA) recommends an HbA1c of between 7.5 and 8.5 % for children less than 6 years. For those between 6 and 12 years, a level of <8 % is recommended while for those >12 years, a level of <7.5 % is considered optimal.

Glycaemic control in type 1 diabetics in Dar es Salaam was previously found to be very poor with mean HbA1c of 10.65 %. This was in a survey carried out on children and adolescents aged 5 to 18 years at the Muhimbili National Hospital clinic for diabetes [3]. Other studies from the East African region have also demonstrated poor control. In a Kenyan study, the median HbA1c was found to be 11.1 % in children and adolescents with T1DM [5].

Various risk factors and challenges have been described that are associated with glycaemic control. Some of these include socio-demographic variables such as age of the child, socioeconomic status, and family structure [6–8]. Other diabetes related factors such as duration of diabetes, adherence and caregiver involvement in the child’s care have also been significantly associated with glycaemic control [9, 10] Most of these studies were done in Europe and North America and very little data exists about risk factors in low resource settings in sub – Saharan Africa.

The aim of this study was to identify the factors associated with glycaemic control in children and adolescents with diabetes in Dar es Salaam. This will help to implement targeted interventions to improve control in this population and prevent chronic complications.

## Methods

This cross-sectional study was carried out over a 6 month period from October 2010 to March 2011 at the diabetic clinic for children and adolescents at Muhimbili National Hospital in Dar es Salaam. This clinic runs once every week on Mondays since its inception in 2007. Patients are managed by a team of 2 nurses, 2 pediatricians trained in endocrinology and 3 support staff.

All children and adolescents attending the clinic are provided with insulin at no cost through the International Diabetes Federation (IDF), Life for a child program. In addition, they are provided with glucose monitors, strips and diaries for self-monitoring and recording of blood glucose at home. They are encouraged to monitor their glucose at least 3 times a week at specified times and to record the readings in their diaries.

HbA1c is measured for all patients every 3–6 months and the screening for retinopathy, nephropathy and neuropathy done yearly. Patients and guardians are also given diabetes education and advice on appropriate nutrition.

All children and adolescents up to and including 18 years of age were eligible for inclusion in the study if consent was provided by the parent/guardian or assent by the adolescent. Those who were newly diagnosed or who had been on treatment for less than 3 months were excluded. All children actively attending the diabetes clinic were recruited in the study, therefore sample size was not calculated.

Data was collected using a structured questionnaire. For children older than 10 years, one section of the questionnaire assessing diabetes knowledge was administered separately to the child and parent/guardian. The questionnaire was translated into the local language, Kiswahili and had the following 3 components:

1. Socio demographic and background information
2. Diabetes knowledge of children/adolescents and caregivers: This was assessed by use of the Michigan Diabetes Research and Training Center’s brief diabetes knowledge test. [11] This was a multiple choice questionnaire which was modified, translated and back translated into Kiswahili. A total of 14 questions out of the 23 item questionnaire were used. Nine questions were omitted because they were not applicable to our patient population. The results of the test were scored based on percentage of correct responses.
3. Diabetes related practices:

a. Insulin adherence: This was determined by the number of insulin doses missed in the last 1 week and was graded as follows:

Good: no missed doses
Average: between 1 and 3 missed doses
Poor: >3 missed doses

b. Dietary adherence: This was assessed by use of a 24 h dietary recall. All meals and snacks eaten in the last 24 h prior to the clinic visit were documented and adherence was graded based on a score derived from the dietary guidelines given at the clinic:

**Table:** 

Component:	Score
Meal frequency:	
3 meals and 3 snacks OR 3 meals and 2 snacks	4
3 meals and 1 snack	3
3 meals only	2
< 3 meals	1
Meal content: (lunch and dinner)- score each meal separately then divide by 2	
All components (carbohydrates, proteins, vegetables)	4
Carbohydrates and vegetables	3
Carbohydrates and protein	2
Only carbohydrates	1
Forbidden foods: Each time a forbidden food is consumed	−1
Interpretation:	
Maximum score: 8	
< 4 – poor	
4–6 – average	
> 6 – good	

c. Blood glucose monitoring (BGM) adherence: This was graded as follows

Good: ≥3 times a week
Average: 1–2 times a week
Poor: none

d. Caregiver involvement in diabetes related tasks: This was assessed by using a scale graded as minimal, moderate or optimal involvement. This was modified from the scale used in the study by Anderson et al. [7]

Involvement in insulin injections was determined by the number of doses in the last 24 h injected or supervised by the caregiver.

**Table:** 

Involvement	Number of injections supervised in the last 24 h
Minimal	None
Moderate	1 - for those on a 2 injection regimen
	1 or 2 - for those on a multiple dose regimen
Optimal	All injections

Involvement in BGM was determined by the degree of participation of the caregiver in the task

**Table:** 

Involvement	Participation of caregiver
Minimal	No participation
Moderate	Reminds the child to check blood glucose
	Enters glucose level in the diary
	Asks child about the blood glucose level
Optimal	Sets up the meter
	Does the finger prick
	Supervises the task

e. Insulin storage: this was classified as refrigeration, storage in a pot of cold water or storage at room temperature.

Glycaemic control was determined by measurement of glycosylated hemoglobin levels (HbA1c). This was determined on all children and adolescents from January to March 2011 using a DCA 2000+ analyzer. Socio demographic characteristics and diabetes specific variables were summarized using frequency distribution tables. Mean and median was calculated for continuous data. Association between variables was tested by use of chi square and fishers’ exact tests. Means were compared using students’ T- test and ANOVA (analysis of variance). Multivariate linear regression analysis was performed to determine factors independently associated with glycaemic control. A *p* value of less than or equal to 0.05 was considered statistically significant. Epi Info software version 3.5.1 and SPSS version 16 were used for data processing.

### Ethical issues

Ethical clearance was granted by the Muhimbili University of Health and Allied Sciences (MUHAS) Institutional review Board (www.muhas.ac.tz) and the Muhimbili National Hospital. Children who were able to provide assent were requested along with consent from care-takers.

After the data was collected and results obtained for HBA1c, participants were further counseled and educated on the same. Hurdles to good glycaemic control were individualized and measures to improve instituted after the data was collected.

## Results

During the study period (October 2010 to March 2011), 90 children and adolescents aged up to 18 years inclusive were registered at the clinic. Of these, 5 did not attend clinic during the study duration, 5 had diabetes for less than 3 months and 2 did not consent. Seventy-eight were included in the study. Three of these did not come for the HbA1c testing and were excluded from final analysis.

The mean age was 13.4 ± 3.9 years (median: 14.1, range: 3.5–18.9 years) with an almost equal sex distribution (50.7 % males). Thirty eight (50.7 %) children were >14 years of age. Most of the children (68 %) were on a multiple daily insulin injection regimen. The socio demographic and diabetes specific characteristics of study participants are summarized in Table 1.

**Table 1:** Socio demographic and diabetes specific characteristics of the participants

Characteristics	Frequency		Median (range)
	*N* = 75	%	
Age (years)			14.1 (3.5, 18.9)
<10	17	22.7	
10–14	20	26.7	
>14	38	50.7	
Sex			
Male	38	50.7	
Female	37	49.3	
Family structure			
2 parent family	47	62.7	
Single parent	17	22.7	
Not living with parents	7	9.3	
Orphan	4	5.3	
Primary caregiver			
Mother	50	66.7	
Father	10	13.3	
Sibling	5	6.7	
Other	10	13.3	
Caregiver education			
No formal education	7	9.3	
Primary	39	52.0	
Secondary	13	17.3	
Tertiary	16	21.3	
Duration of DM (years)			3.17 (0.33,11.2)
<1	10	13.3	
1–5	46	61.3	
>5	19	25.3	
Insulin dose (units/kg body wt/day)			1.0 (0.24,2.0)
Insulin regimen			
2 daily injections	24	32.0	
Multiple daily injections	51	68.0	

The mean HbA1c was 11.1 ± 2.1 %. There was no difference in the mean HbA1c between males and females. However, children in the younger age group (<10 years) had a significantly lower mean HbA1c as compared to the older children.

Children with mothers as the primary caregivers had a significantly lower mean HbA1c as compared to those whose caregivers were a father, a sibling or another family member. The family structure of the patient and the education level of the caregiver were not significantly associated with glycaemic control. (Table 2)

**Table 2:** Factors associated with Glycaemic control

Characteristics	Mean HbA1c (% ± 2SD)	*P* value^a^
Age group		
< 10 years	9.8 ± 2.0	0.022^b^
10–14 years	11.5 ± 2.1	
> 14 years	11.4 ± 2.0	
Sex		
Male	11.0 ± 2.3	0.907
Female	11.1 ± 2.0	
Family structure		
2 parent family	10.9 ± 2.1	0.377
Other	11.3 ± 2.2	
Primary caregiver		
Mother	10.7 ± 2.1	0.025^b^
Other	11.8 ± 2.0	
Caregiver education		
None/Primary	11.3 ± 2.1	0.26
Secondary/tertiary	10.7 ± 2.1	

^a^Calculated using students’ *t* test and ANOVA

^b^Statistically significant

Shorter duration of disease was associated with significantly lower HbA1c levels. Children who had T1DM for less than 1 year had a mean HbA1c that was 2 % lower than those with disease for a longer duration (*p* value = 0.011).

The insulin regimen used did not influence the HbA1c. Children who were on the multiple daily insulin injection regimens had an equal mean HbA1c as compared to those who were on 2 daily injections.

The mean diabetes knowledge score of parents/caregivers was 70 ± 15 % while assessment of diabetes knowledge of the adolescent revealed a similar mean score of 70.3 ± 15.9 %. Simple linear regression analysis found diabetes knowledge of caregivers to be significantly associated with HbA1c. (r2 = 0.07, *p* value = 0.036). Knowledge of children was not associated with HbA1c (*p* value = 0.868)

Adherence to the insulin regimen was found to be good in majority of study participants (68 %). Reasons cited for poor adherence included forgetfulness and inconvenience in injecting insulin at school and other public places.

Forty-eight percent of children reported good adherence to the BGM regimen prescribed at the clinic while 24 and 28 % reported average and poor adherence respectively. The most common reason for poor adherence was unavailability of BGM strips.

**Fig. 1:**
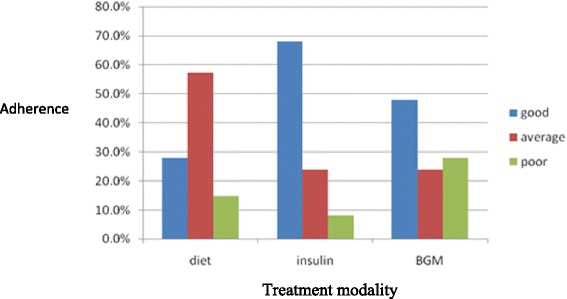
Adherence to diet, insulin and blood glucose monitoring

Dietary adherence was average in most of the patients (57.3 %). Difficulty in reducing carbohydrate quantity was a common problem encountered by most adolescents. Adherence is summarized in Fig 1.

The mean HbA1c did not differ with adherence to diet and insulin. Adherence to BGM regimen was associated with lower mean HbA1c in those with average adherence as compared to those with poor adherence. (10.1 ± 2.1 vs 11.7 ± 2.1, *p* value 0.061)

Caregiver involvement in diabetes related tasks of the child varied with the type of task. In the task of insulin injection, 44 % of caregivers exhibited optimal involvement as compared to only 29.3 % in the task of BGM. Caregiver involvement in diabetes related tasks was not significantly associated with HbA1c.

Insulin was reported to be stored in the fridge by 65.3 % of participants; either their own or a neighbors. Thirty-two percent stored it in a pot of water while the remaining 2.7 % kept the vials at room temperature. The difference in the mean HbA1c between those who stored insulin in the fridge and in a pot of water was not statistically significant (11.1 vs. 11.1 %, *p* value = 0.85).

To determine predictors of glycaemic control, a multivariate regression analysis was performed using variables that had a significant association with HbA1c (*p* < 0.05) and those that approached statistical significance (*P* < 0.1) in bi-variate analysis The variables tested in the multivariate analysis were: age of the child, primary caregiver, diabetes knowledge of caregiver, duration of disease and adherence to BGM regimen. Results of the multivariate analysis are shown in Table 3.

**Table 3:** Multivariate analysis of the significant factors associated with glycaemic control

Variable	Beta (standardized coefficients)	*P* value
Age of patient	0.404	0.002 ^a^
Duration of DM	−0.144	0.184
Primary caregiver (mother/other)	0.312	0.005 ^a^
Adherence to BGM regimen (poor/good)	0.016	0.908
Adherence to BGM regimen (poor/average)	−0.234	0.03 ^a^
Diabetes knowledge of caregiver	−0.086	0.439

^a^Statistically significant

*DM* diabetes mellitus, *BGM* blood glucose monitoring

The independent variables in the model predicted 33.2 % of the variance in HbA1c

(R^2^ = 0.332, *p* value = 0.000)

## Discussion

The mean glycaemic control in this study was 11.1 ± 2.1 %. The factors that were significantly associated with glycaemic control were age, adherence to blood glucose monitoring regimen and primary caregiver.

There is a marginal increase in the mean HbA1C from the mean of 10.65 ± 2.09 which was reported at the same center by Majaliwa et al. in 2006 [3]. This increase is alarming because the previous study was done in the setting of very poor insulin supplies and unavailability of BGM at home. Despite major progress in the availability of insulin and blood glucose monitors, glycaemic control showed no improvement. This points to the existence of other underlying factors which have yet to be identified that are contributing to poor glycaemic control. Results of the DCCT demonstrated that every 1 % decrease in HbA1c results in a 40 % reduced risk of developing retinopathy. The importance of lowering the level of HbA1c in our population of type 1 diabetics cannot be overemphasized [4].

Very few studies have been done in Africa documenting glycaemic control. A Kenyan study by Ngwiri also showed poor glycaemic control with a median HbA1C of 12.1 % [5]. A study by Pillay et al. from South Africa documented a mean HbA1C of 9.7 % in children with T1DM aged 6–10 years [12]. This is similar to the mean HbA1c in children <10 years in the current study (9.83 %). Younger age has consistently been found to be associated with better glycaemic control in several studies [6, 7]. This may be attributed to more parental supervision in this age group.

Adolescents with T1DM have been reported to have poor glycaemic control [10]. The poor glycaemic control with increasing age as demonstrated in the current study may be due to the onset of puberty and the hormonal changes that happen at this age. Elevated levels of growth hormone and reduced level of insulin like growth factor may contribute to weight gain in adolescent girls. This in turn, results in altered eating habits and missed insulin doses. In addition, psychological and social stressors in this age group may also contribute to poor glycaemic control. This is thought to be mediated by stress hormones such as cortisol and catecholamines which increase blood glucose. Reduced parental supervision and more independent control of the disease may also contribute to worsening control.

The role of the family in the management of T1DM in children and adolescents has been described. In a study by Thompson et al., children from single parent families had a significantly higher HbA1c as compared to those from 2 parent families [13]. This was also demonstrated in a study by Araujo et al. in Argentina [8]. In the current study however, children from 2 parent families did not have significantly better glycaemic control. This could be explained by the role that parents play in caring for a sick child in the African societies, where it is the primary responsibility of the mother to care for sick children. Indeed this was demonstrated by the significantly lower mean HbA1c (10.8 %) in children whose primary caregiver was the mother as compared to 12.12 % in those who had a primary caregiver other than the mother.

The mean duration of diabetes in our population was 3.74 years. Children with duration of <1 year had significantly better mean HbA1c compared to those with duration >1 year. This is a common finding in newly diagnosed diabetics due to the “honeymoon effect”; this is a period of relatively good glycaemic control that occurs due to residual beta cell function in the 1st year following diagnosis and initiation of treatment. In multivariate analysis, however, duration of DM did not significantly predict HbA1c. This is contrary to many studies which have reported diabetes duration as a significant predictor of HbA1c [6, 7, 14] The lack of association between duration and HbA1C in our study could be explained by the overall poor glycaemic control which may mask any impact of duration on the disease.

In our study, majority of the children (68 %) were on a multiple insulin injection regimen. This is currently one of the recommended modes of treatment of T1DM, the other being the continuous subcutaneous insulin infusion (CSII) using a pump [15]. However, the mean HbA1c in this group did not differ from those on the conventional 2 daily insulin injection regimen. This finding is surprising given the important role of prandial insulin in lowering blood glucose. It could be explained by the suboptimal adherence to the insulin regimen and missing of the lunchtime insulin dose. Around 30 % of children reported missing one or more doses of insulin per week due to inconvenience in injecting in public places.

Diabetes education is a key component in the management of T1DM. Education of both the caregiver and the child is important to ensure adherence to the multiple diabetes related tasks. In the current study population the mean parental knowledge score was 70 ± 15 %. This indicates a significant deficit in knowledge.

Higher parent diabetes knowledge scores were associated with a significantly lower HbA1c. These results were similar to those in a study by Butler et al. [16] where higher parental diabetes specific knowledge was significantly correlated with a lower HbA1c. In another study by Stallwood et al. [17], parental diabetes knowledge was tested using the Michigan Diabetes Research and Training Centre’s brief knowledge test using all the 23 items in the questionnaire unlike the current study where we used 14 out of 23. The mean parental score in that study was 74 % and higher scores were correlated to lower HbA1c’s.

The mean diabetes knowledge score of the youth was similar to that of the parents but did not show association with age or with HbA1c. This finding emphasizes the key role of optimizing parental knowledge to achieve better control in the child.

There was varying adherence to the different treatment modalities. Best adherence was observed to the insulin regimen (68 %) followed by BGM (48 %) and lastly diet (28 %). This reflects the underlying importance patients given to insulin administration and the relative complacency to the other modes of treatment. The lack of a regular supply of BGM consumables could also contribute to poor adherence

Overall, better adherence in the diet and insulin regimen domains did not translate to better glycaemic control. However, adherence to BGM was significantly associated with HbA1c in multivariate analysis.

The adherence and glycaemic control link has been examined in several studies including a meta - analysis carried out by Hood et al. [9] This meta - analysis demonstrated an effect size of −0.28 (CI −0.32, −0.24). This implies that better adherence leads to lower HbA1c. This meta-analysis did not demonstrate any association between socio-demographic or disease specific variables on the overall effect size. A study by Mehta et al. examining dietary adherence showed that a higher dietary score was associated with lower HbA1c [18]. The absence of significant association between HbA1c and adherence to insulin and diet in our study may be explained by the method used to assess adherence which was primarily self-reporting. This method is prone to recall bias and misreporting and this may have resulted in falsely elevated adherence levels. Other studies have used caloric counting to assess dietary adherence; this however is not applicable in our population. BGM adherence can be determined by data from blood glucose meter downloads. In the current study population however, this was not practical because most patients did not carry meters to the clinic during visits.

The recommended method of insulin storage is refrigeration at a temperature of 2 - 8 °C for unopened vials of insulin. Open vials may be kept at room temperature but must be used within 1 month. Due to the low availability of refrigerators, 32 % of our patients with diabetes store their insulin in a pot of water which is kept cool by use of charcoal to absorb heat. The method of insulin storage did not significantly affect glycaemic control. This may be explained on the basis of the limited insulin stocks available to the patients, which usually do not last more than 1 month.

Multivariate analysis of variables associated with glycaemic control found 3 main predictors of HbA1c: age, primary caregiver and adherence to BGM regimen

The limitations in this study were the cross sectional design which is not the best design to demonstrate relationships between variables and the small study population. Effort was made to recruit all eligible participants who met inclusion criteria. Despite the small number, we believe the results of this study provide the much needed data to improve glycaemic control in resource limited settings like ours.

## Conclusions

In conclusion, we found that metabolic control is very poor in our population of type 1 diabetics with overall poor adherence. Factors associated with poor control include older age, a caregiver other than the mother and poor adherence to BGM. In order to improve metabolic control, more frequent BGM should be encouraged and regular supply of glucose monitoring strips ensured. Emphasis needs to be put on adherence counseling and active participation of caregivers in diabetes related tasks of their children. Close follow up of the adolescents is necessary as this group is the most vulnerable to poor control.

## Abbreviations

T1DM, − Type 1 Diabetes Mellitus; DCCT, − Diabetes Control and Complications trial; ADA, − American Diabetes Association; HbA1c, − Glycosylated Hemoglobin; BGM, − Blood Glucose Monitoring; MUHAS, − Muhimbili University of Health and Allied Sciences.
